# Finite element analysis of patient-specific additive-manufactured implants

**DOI:** 10.3389/fbioe.2024.1386816

**Published:** 2024-05-09

**Authors:** Arman Namvar, Bill Lozanovski, David Downing, Tom Williamson, Endri Kastrati, Darpan Shidid, David Hill, Ulrich Buehner, Stewart Ryan, Peter F. Choong, Reza Sanaei, Martin Leary, Milan Brandt

**Affiliations:** ^1^ RMIT Centre for Additive Manufacture, School of Engineering, RMIT University, Melbourne, VIC, Australia; ^2^ Department of Surgery, St. Vincent’s Hospital, Melbourne, VIC, Australia; ^3^ Stryker, Sydney, NSW, Australia; ^4^ Melbourne Veterinary School, Faculty of Science, The University of Melbourne, Melbourne, VIC, Australia

**Keywords:** bone tumors, patient-specific implants, endoprosthetic reconstruction, biomechanical testing, additive manufacturing, femur

## Abstract

**Introduction:** Bone tumors, characterized by diverse locations and shapes, often necessitate surgical excision followed by custom implant placement to facilitate targeted bone reconstruction. Leveraging additive manufacturing, patient-specific implants can be precisely tailored with complex geometries and desired stiffness, enhancing their suitability for bone ingrowth.

**Methods:** In this work, a finite element model is employed to assess patient-specific lattice implants in femur bones. Our model is validated using experimental data obtained from an animal study (*n* = 9).

**Results:** The results demonstrate the accuracy of the proposed finite element model in predicting the implant mechanical behavior. The model was used to investigate the influence of reducing the elastic modulus of a solid Ti6Al4V implant by tenfold, revealing that such a reduction had no significant impact on bone behavior under maximum compression and torsion loading. This finding suggests a potential avenue for reducing the endoprosthesis modulus without compromising bone integrity.

**Discussion:** Our research suggests that employing fully lattice implants not only facilitates bone ingrowth but also has the potential to reduce overall implant stiffness. This reduction is crucial in preventing significant bone remodeling associated with stress shielding, a challenge often associated with the high stiffness of fully solid implants. The study highlights the mechanical benefits of utilizing lattice structures in implant design for enhanced patient outcomes.

## 1 Introduction

Additive manufacturing (AM) technology has emerged as a promising solution for tumor resection and reconstruction in orthopedics. This technology leverages the capacity to produce complex geometries, coupled with advanced medical imaging, to tailor patient-specific endoprostheses suited to individual medical needs, an achievement unattainable with conventional off-the-shelf prostheses (Shidid et al., 2016). Moreover, the use of AM is expanding to include lattice implants, which reduces implant stiffness, thus stress shielding (Sanaei et al., 2023), and provides enhanced osteointegration by allowing bone ingrowth within the implant structure (Niinomi and Nakai, 2011; Wong, 2016; Park et al., 2021). Given the variability in bone tumor locations within the skeleton and in each bone, the implant location and shape can significantly differ from patient to patient. This introduces considerable variability in the stresses and strains the bone-implant construct will experience which must be taken into account when designing any patient-specific implants. Any errors in modulating the prosthesis modulus and the overall mechanical properties at this stage have the potential to result in either stress shielding, if modulus is too high, or implant failure (fracture), if modulus is too low both of which are very common complications of endoprosthetic reconstruction (Abu El Afieh et al., 2022). The latter will perhaps become even more common as low modulus endoprostheses become more commonplace. The topographic position/location of flanges or fixation screws is another variable that can determine the likelihood of stress concentration and implant failure or aseptic loosening, with the latter being another common reason for surgical failure (Abu El Afieh et al., 2022). Consequently, it becomes imperative to employ numerical modeling to evaluate the behavior and performance of patient-specific additive manufactured implants before surgical intervention. Such modeling allows for the optimization of implant lattice design to not only restore natural bone stiffness but also minimize the occurrence of stress shielding phenomena.

While various finite element (FE) models have been developed to simulate human bones, particularly by extracting bone geometry from CT scans and correlating associated Hounsfield Unit (HU) values to bone material properties (Bessho et al., 2007; Poelert et al., 2012; Basafa et al., 2013; Schermann et al., 2020), only a limited number of studies have employed finite element analysis to simulate the stiffness and behavior of bones implanted with 3D printed implants (Wong et al., 2020; Yan et al., 2020; Liu et al., 2022). Notably, none of these studies have validated their simulation results with experimental data for implanted bones.

In this study, a novel patient-specific FE model is presented to simulate the biomechanical environment of sheep femora that were reconstructed with endoprostheses, following the removal of phantom distal metaphyseal tumors. What sets this study apart is its rigorous validation process, where the FE model’s predictions are compared against experimental data derived from an *in vivo* animal study. This animal study investigated the potential of enhancing tumor resection outcomes by incorporating patient-specific AM implants using surgical robotics (Williamson et al., 2023). The aim of this work was to uncover the disparities between solid and lattice implants using an *in vivo* validated finite element (FE) model to analyze the influence of implant geometry and stiffness on the overall stiffness of implanted sheep femora. The described model is proposed to serve as a biomechanical screening tool in the design stage of custom AM endoprostheses with its main utility to prevent the incidence of acute implant or periprosthetic bone fractures.

## 2 Materials and methods

### 2.1 Animal study

This research was conducted as a part of a larger animal study which was designed to investigate bone ingrowth and the biomechanical properties of sheep femora reconstructed with just-in-time patient-specific additive manufacturing (AM) implants (Shidid et al., 2016), following robot-assisted partial resection of distal metaphysis (Williamson et al., 2023). All animal procedures in this work were approved by the Animal Ethics Committee of the Faculty of Veterinary and Agricultural Sciences, the University of Melbourne (Infonetica # 10442) and were compliant with the Australian Code for the Care and Use of Animals for Scientific Purposes (2013). Readers are referred to Williamson et al. (2023) and Sanaei et al. (2023) for further information on the design and outcome of this study.

Here, we report the results of our *in silico* biomechanical testing completed for 9 sheep using preoperative CT data. For the purpose of validating the model, biomechanical data obtained from animals euthanized at 6 months was utilized.

### 2.2 AM patient-specific implant

This research focused exclusively on the development of a FE model capable of predicting the biomechanical properties of the ovine femur that has been subjected to endoprosthetic reconstruction of its distal metaphysis using patient-specific AM implants. The validation of this model was carried out using experimental data derived from the biomechanical testing conducted as part of the animal study. Two distinct implant types were employed in that study: (i) Solid Implant and (ii) Lattice Implant.

**FIGURE 1:**
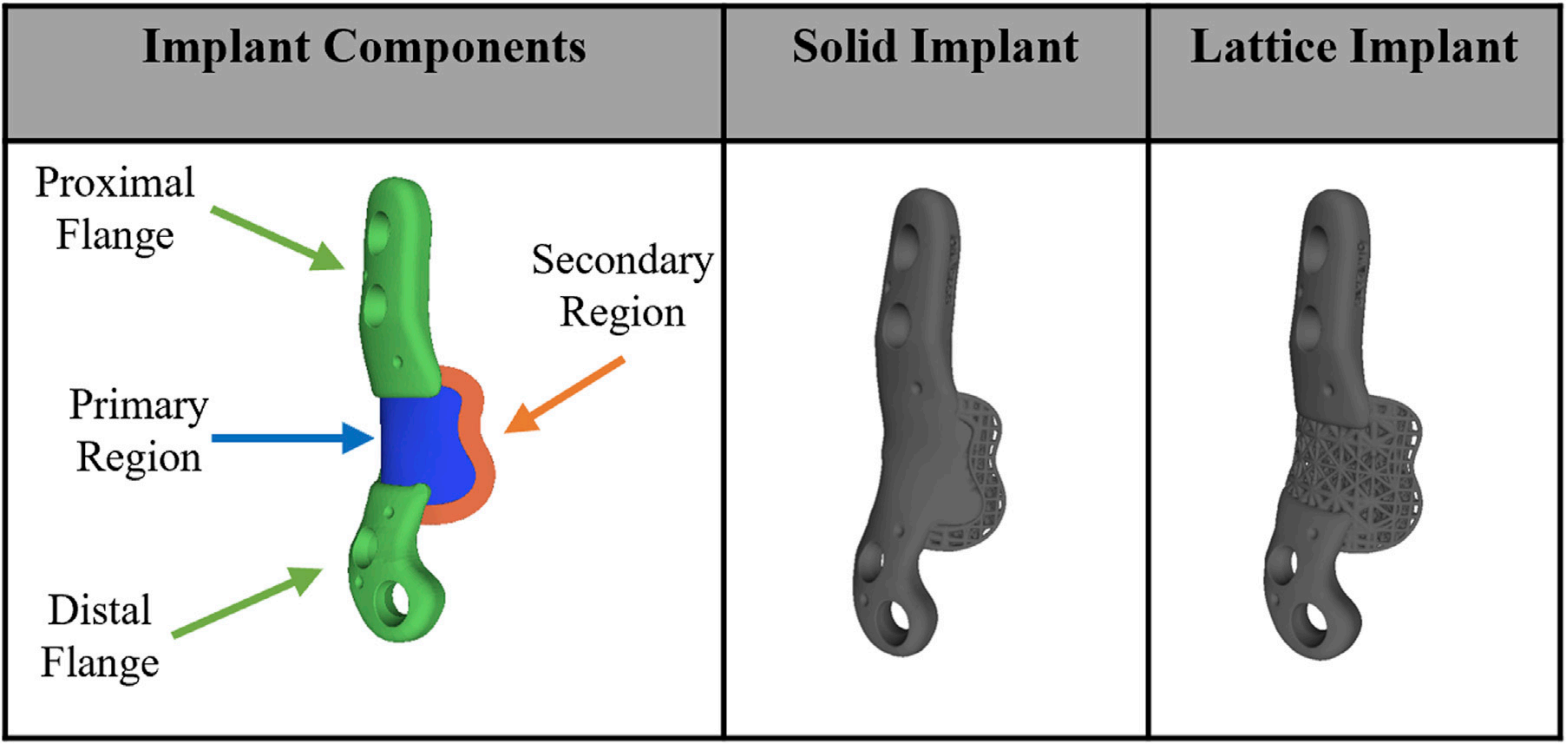
Implant components and overview design of solid and lattice implants. Each implant consists of two flanges, a primary region, and a secondary interfacial region.

These patient-specific implants were designed to conform to the geometry of the sheep femora. Six sheep received the solid implant in their distal femoral metaphysis and three received the lattice type. Each implant was manufactured using Laser Beam Powder Bed Fusion (LB-PBF) technology (SLM 125, SLM solutions) and constructed from Ti6Al4V ELI powder (SLM solutions). The implants featured distinct components, including flanges, primary regions, and secondary regions, as illustrated in Figure 1.

In the secondary region of both implant types, a Face-Centered Cubic with Z-Strut (FCCZ) lattice structure was employed. This structure featured a unit cell measuring 2 × 2 × 2 mm, with the Z-struts aligned along the primary anatomical axis of the femur. The primary distinction between the solid and lattice implants resided in the composition of the primary region. In the solid implant, the primary region consisted of solid Ti6Al4V, while in the lattice implant, it was filled with an FCCZ lattice structure employing a unit cell of 4 × 4 × 4 mm. For further details regarding the design and manufacturing of these AM implants, refer to the publication by Shidid et al. (2016).

### 2.3 3D FE model

**FIGURE 2:**
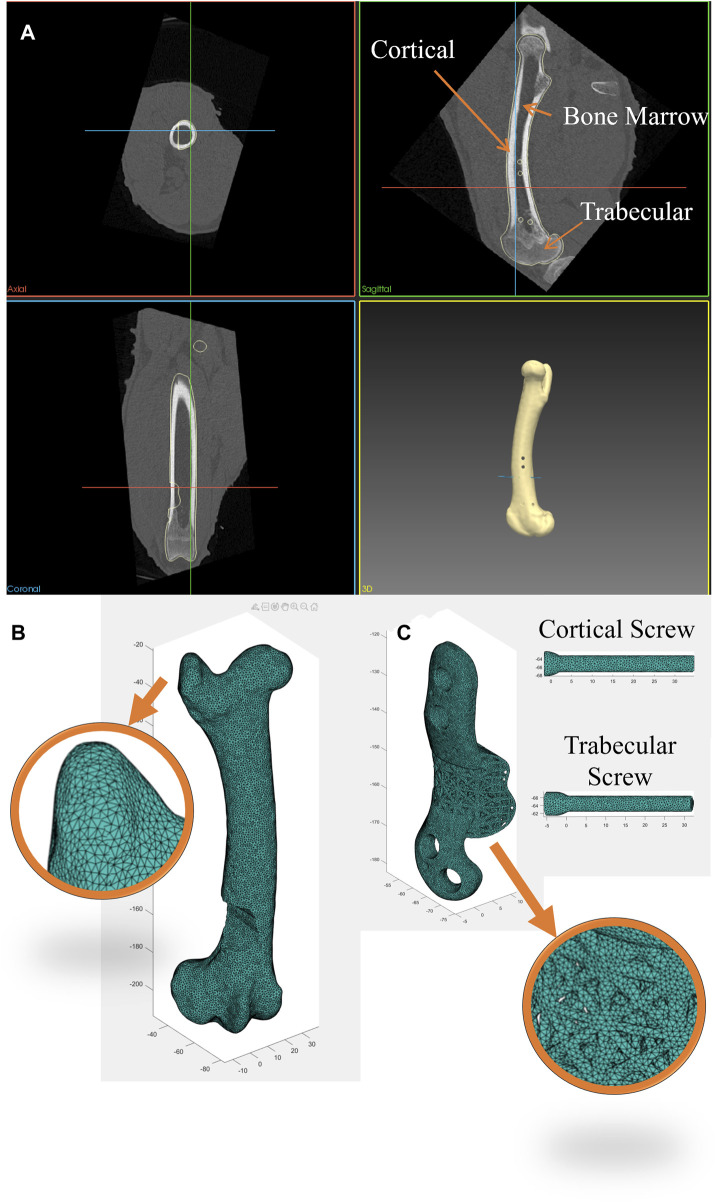
**(A)** Segmentation of bone CT images was performed using a custom image and mesh processing software. **(B)** 3D tetrahedral finite element mesh of the bone following phantom tumor resection generated from the CT dataset using the MATLAB toolbox, Iso2mesh, and **(C)** 3D tetrahedral finite element mesh of the implant and screws (cortical and trabecular screws) generated in the MATLAB toolbox, Iso2mesh.

In this study a FE model was employed to simulate the biomechanical behavior of the right ovine femur following implantation, with the left femur serving as the control. Pre-operative CT scans of the femora were used to acquire detailed anatomical and density information. Custom image and mesh processing software was employed for CT image segmentation (Stryker, United States of America). This process enabled the creation of a 3D model of each bone, which was then exported as an STL file. The STL file, representing the segmented bone geometry, was further processed to create a 3D tetrahedral finite element mesh (Figure 2). To achieve this, Iso2mesh, an open-source MATLAB mesh generation and processing toolbox developed by a research group at Massachusetts General Hospital, was utilized (Fang and Boas, 2009). The tetrahedral mesh was then imported into Abaqus software for subsequent analysis. 3D volume meshes of the implant and screws were also generated similarly from their STL files (Figure 2C).

To replicate each bone’s heterogeneous material properties, material density values were derived from the CT datasets and assigned to the corresponding elements of each bone mesh. Past research (Wirtz et al., 2000; Peng et al., 2006) has demonstrated that bone material properties can be estimated from Hounsfield Unit (HU) values in CT scans. The bone apparent density for each element was calculated using a linear relationship (Eq. (1)). It was assumed that cortical bone has an apparent density of 2.0 g/cm^3^ corresponding to the maximum HU values found in CT scans (∼2000–2,200), while water has an apparent density of 1 g/cm^3^ with an HU value of 0.

$$\rho =\frac{HU}{{HU}_{\max}}+1$$ (1)

Subsequently, bone Young’s modulus was calculated for each element from the bone apparent density using Eq. (2) (Wirtz et al., 2000; Peng et al., 2006) to classify cortical bone, trabecular bone and bone marrow according to the associated $HU_{\text{mean}}$ .

$$\begin{array}{c} HU_{\text{mean}}\geq 1000\;E=2065\;\rho^{3.09},\nu =0.3\text{ Cortical bone}\\ 300\leq HU_{\text{mean}}<1000\;E=1904\;\rho^{1.64},\nu =0.3\text{ Trabecular bone}\\ HU_{\text{mean}}<300\;E=20\;MPa,\nu =0.499\text{ Bone marrow} \end{array}$$ (2)

Where *E* is the Young’s modulus in MPa and ν is the Poisson’s ratio. Lastly, the elastic properties of each element were mapped to the bone using a predefined field in Abaqus. A mesh convergence study was conducted for the bone model, and the results found a mesh with a global element size of 1 mm to be sufficient for convergence (approximately 650,000 linear tetrahedral elements, C3D4).

Implants and screws were modeled using linear tetrahedral elements (C3D4) and a linear elastic model with Young’s modulus of 110 GPa and Poisson’s ratio of 0.3 to represent SLM Ti6Al4V (Xiao and Song, 2018). A general contact interaction model was established to account for interactions between all surfaces of the bone, implant, and screws. This model encompassed both hard normal contact and tangential contact, with a friction coefficient of 0.14.

Four screws were used by the surgeon to secure the implant to the bone. This included two 3.5 mm diameter cortical screws (Stryker AxSOS 3 3.5 mm cortex screw, self-tapping, T15 drive, Stryker, United States of America) and two 4 mm diameter trabecular screws (Stryker AxSOS 3 4.0 mm cancellous screw, full thread, self-tapping, T15 drive, Stryker, United States of America). As all screws were self-tapped into the bone, they underwent corresponding preloading. The axial force resulting from preloading was estimated using theoretical formulas as described in (Seneviratne et al., 2001) and applied to the screws in the FE model through negative thermal expansion (thermal contraction) using a predefined field in the initial step in Abaqus. This action effectively introduced an equivalent axial preloading in the screws.

It is important to note that while the thread geometry of the screws was not explicitly modeled in this study, the thread portions of the screws within the bone were fixed to the adjacent bone elements using a tie constraint in Abaqus to reflect their real-world behavior.

### 2.4 Biomechanical testing setup

**FIGURE 3:**
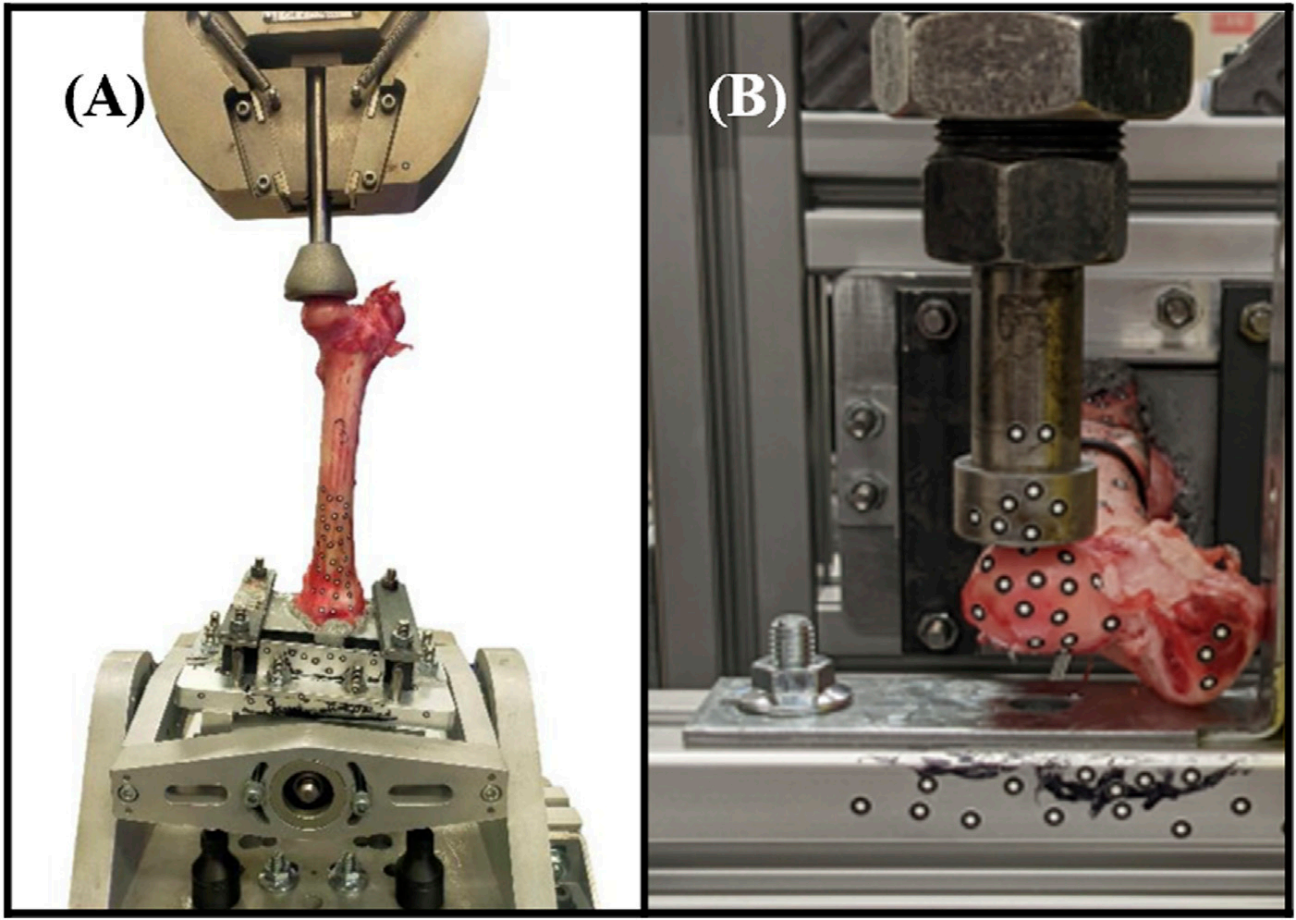
Biomechanical testing setups. **(A)** Axial compression testing comprised applying load to the mechanical axis of the femur. **(B)** Torsion testing comprised applying an offset load to the femoral head (part of the hip joint).

Axial compression and torsion tests were conducted on both the implant-fitted (right) and control (left) femora using an Instron Machine (Instron 5969, Instron Corporation, Massachusetts, MA, United States of America), as shown in Figure 3. In all tests, the distal extremity of all bones was firmly fixed in a plastic box using a hybrid polyurethane filler (Dunlop Ardit Liquid Crack Filler). In compression tests, a downward displacement of 1.5 mm was applied to the femoral head, aligned with its mechanical axis (approximately 6°–8° offset from its femoral axis). This displacement was applied at a constant rate of 0.15 mm/min, providing quasi-static loading. For torsion tests, the bone was positioned horizontally, with the lesser trochanter supported against a bracket. A downward displacement of 1.5 mm, applied at a rate of 0.15 mm/min was used to induce torsional forces in the bone. Each of these tests was repeated three times to ensure the reliability and consistency of the results. Similarly, in the FE simulations, a fully constrained boundary condition (Encastre condition) was applied up to 10 mm from the bottom of the distal extremity, while a downward displacement of 1.5 mm in the specified direction was applied to the femoral head using rigid applicators, modeled from real geometry.

### 2.5 Statistical analysis

Correlation between the FE predicted values and the actual experimental values was tested using the non-parametric Spearman’s test in IBM SPSS Statistics version 29 (IBM Corp^©^, Armonk, NY, United States of America).

## 3 Results

### 3.1 FE model validation

**FIGURE 4:**
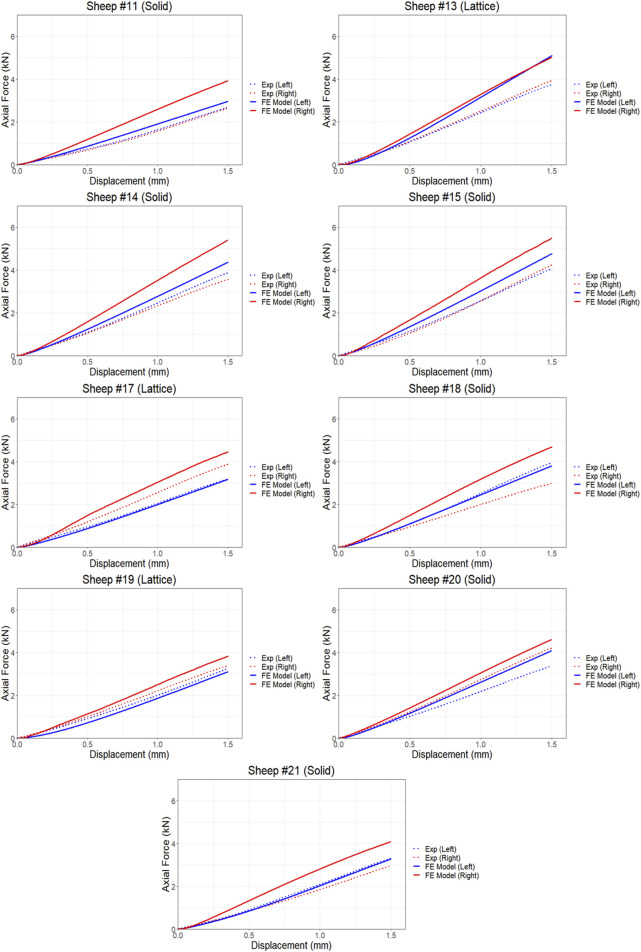
Comparison of force and displacement curves obtained from FE simulations and experiments results using compression testing.

**FIGURE 5:**
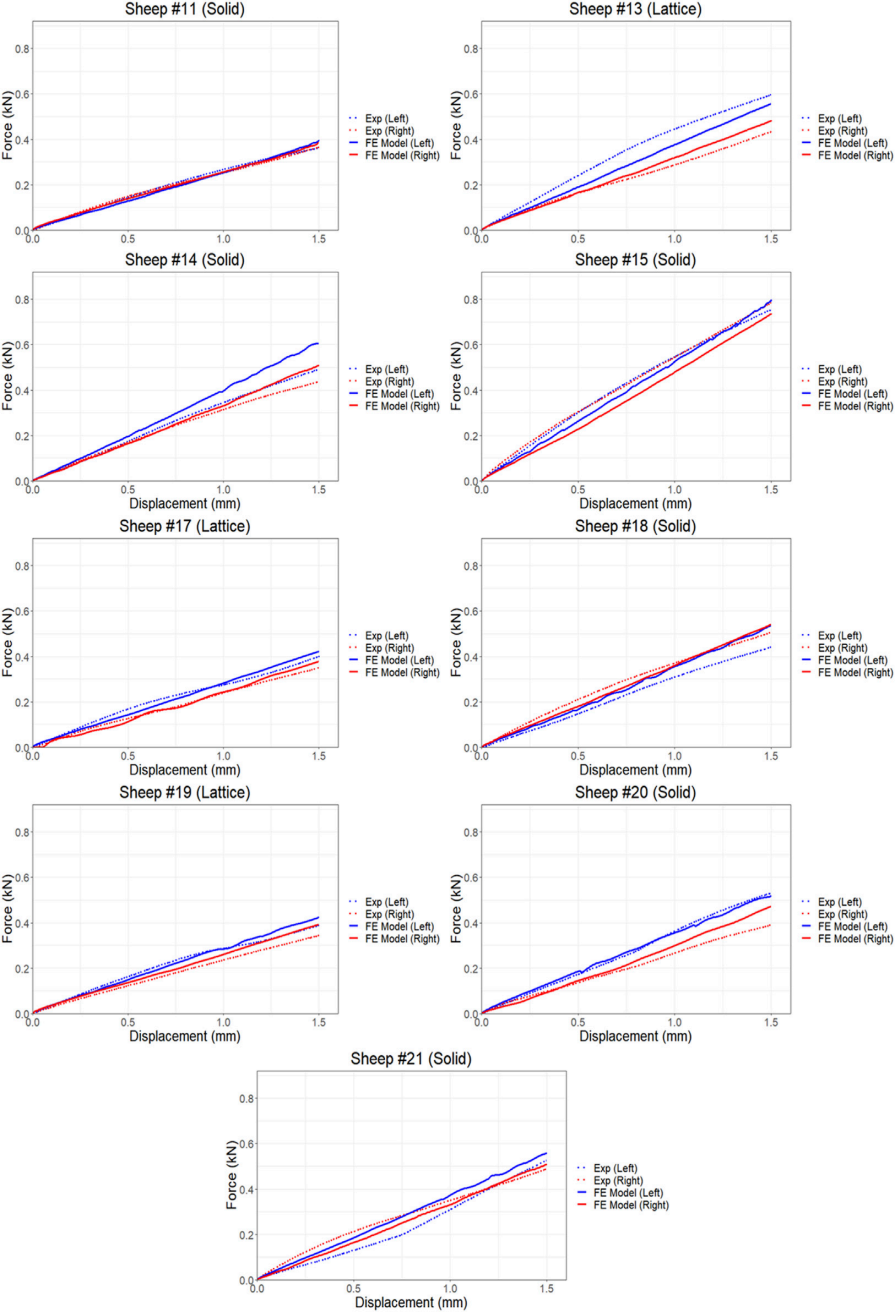
Comparison of force-displacement curves obtained from FE simulations and experiments results using torsion testing.

The proposed FE model of each bone was validated against experimental data obtained from the control bones (left femora, no implant). To model the implanted bones, a 3D representation of the implant and screws was incorporated within the bone model utilizing tetrahedral elements. The results demonstrate strong agreement between the simulations and the experimental data derived from both the compression and torsion tests at 6 months post-implantation. Table 1 presents the disparities in calculated axial and torsional stiffnesses between the FE model’s predictions and the experimental data for the control bones (left femora, no implant) with an average difference of approximately 12%, indicating the good accuracy of the proposed FE model. The axial and torsional stiffnesses were simply calculated by fitting a linear regression to the plots ( $R^{2}>0.98$ ). Figures 4, 5 illustrate the force and displacement curves for all 9 sheep from the animal trial and comparing the experimental and the simulated results for both the implanted and control femora under compression and torsion loadings. Statistical analysis showed that overall, there was a correlation between the FE predicted values and those that were measured experimentally for the axial compression study, r (df) = 0.555, *p* = 0.017 and a very strong correlation between the two methods for the torque study, r (df) = 0.962, *p* < 0.0001. Similarly, a stronger correlation was observed between the simulated and actual values for the torsional loading study compared to compression loading when control and implanted bones were analyzed separately (Table 2).

**TABLE 1:** Comparison of axial and torsional stiffnesses between the FE model and experiments in control (left femora, no implant) and implanted bones (right femora, solid/lattice implant).

Sheep No	Axial stiffness (kN/mm)						Torsional stiffness (kN.mm/rad)					
	Control bone (left femur)			Implanted bone (right femur)			Control bone (left femur)			Implanted bone (right femur)		
	Experiment	FE model	Diff (%)	Experiment	FE model	Diff (%)	Experiment	FE model	Diff (%)	Experiment	FE model	Diff (%)
#11 (Solid)	1.79	1.97	9.9	1.78	2.66	50.0	398	420	5.5	375	403	7.5
#13 (Lattice)	2.59	3.38	30.5	2.73	3.42	25.2	652	604	7.4	445	515	15.7
#14 (Solid)	2.65	2.9	9.5	2.45	3.64	48.6	545	661	21.3	474	553	16.7
#15 (Solid)	2.76	3.16	14.6	2.92	3.73	27.8	816	857	5.0	819	790	3.5
#17 (Lattice)	2.16	2.1	2.6	2.65	3.1	16.8	406	453	11.6	376	410	9.0
#18 (Solid)	2.74	2.55	7.1	2.04	3.22	58.1	496	576	16.1	538	583	8.4
#19 (Lattice)	2.19	2.03	7.5	2.34	2.6	11.2	410	459	11.9	369	417	13.0
#20 (Solid)	2.29	2.73	19.3	2.91	3.12	7.1	594	566	4.7	418	503	20.3
#21 (Solid)	2.3	2.16	6.2	2	2.84	42.2	577	602	4.3	494	553	11.9

**TABLE 2:** Correlation between the simulated and experimental findings.

Experimental group	Axial stiffness		Torsional stiffness	
	r (df)	*p*-value	r (df)	*p*-value
Overall (Control & Implanted Femora)	0.555 (16)	0.017	0.962 (16)	<0.0001
Control Bone (Left Femur)	0.783 (7)	0.013	0.833 (7)	0.005
Implanted Bone (Right Femur; Solid & Lattice)	0.650 (7)	0.058	0.946 (7)	<0.001

**FIGURE 6:**
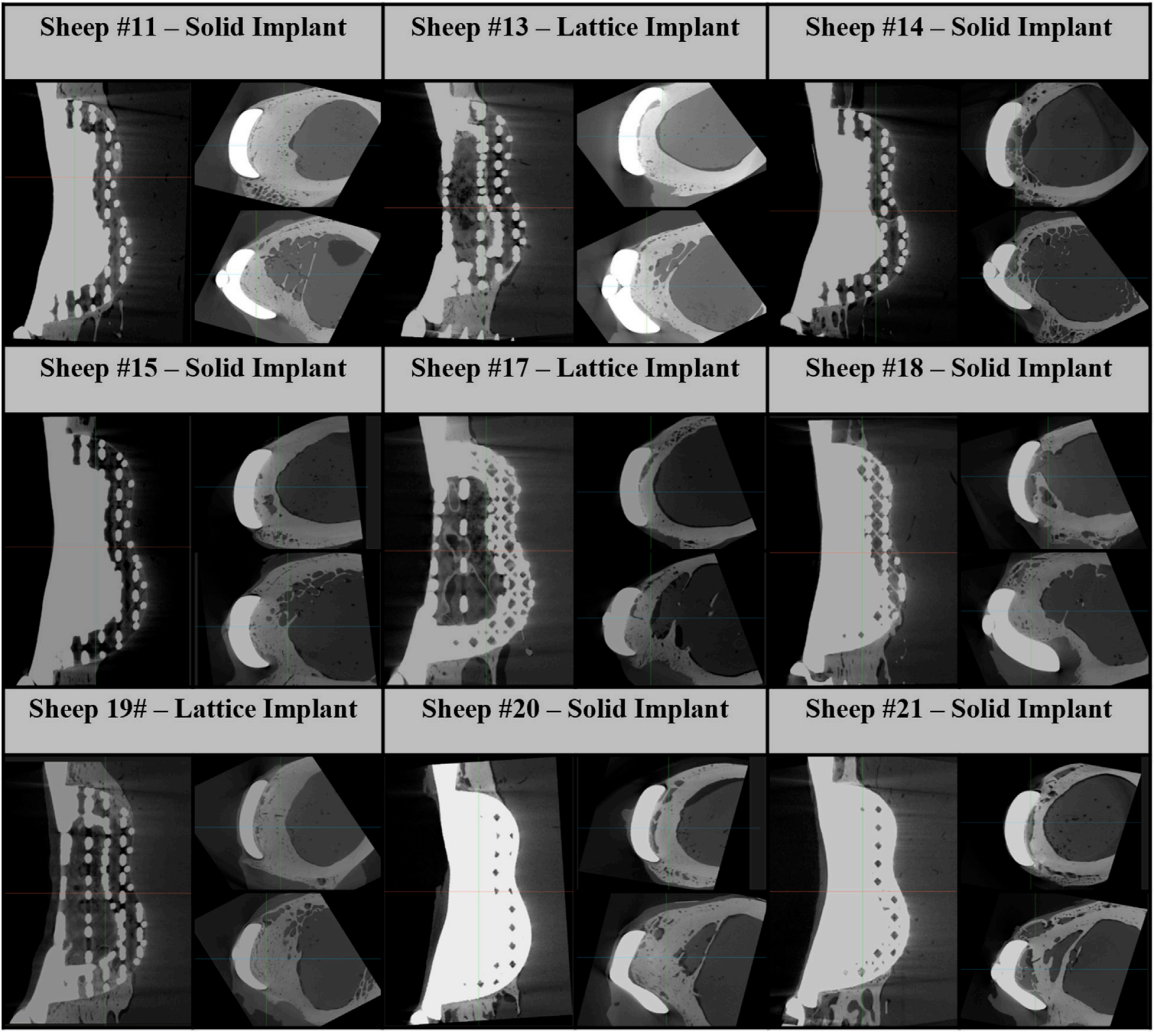
Micro-CT images of 6-month implanted femur bones. These images consist of one sagittal image and two axial images below the proximal and distal flanges.

We found average differences of 18% and 39% in axial stiffness between the FE model and experimental results within the lattice and solid implant cohorts respectively. Similarly, the average differences in torsional stiffness were noted to be 12.5% and 11% for the lattice and solid implant cohorts respectively in that order (Table 1). These results suggest that while there was generally good agreement between the FE model’s predictions and the experimental data, some discrepancies were also present. Micro-CT scans revealed substantial structural remodeling within the implanted bones after a 6-month survival period. Notably, the observation of significant bone resorption in the vicinity of the solid implanted bones contributed to larger discrepancies between the simulation and experimental results, particularly when compared to the lattice implanted bones. Figure 6 visually portrays the structural remodeling surrounding the implant in the bones from the animal trial. These images depict that the bones with solid implants experienced more pronounced remodeling and resorption beneath the implant flanges. Conversely, bones with lattice implants demonstrated more promising signs of bone ingrowth within the implant, suggesting the potential for a more favorable outcome in promoting osteointegration.

### 3.2 High stress zones

**FIGURE 7:**
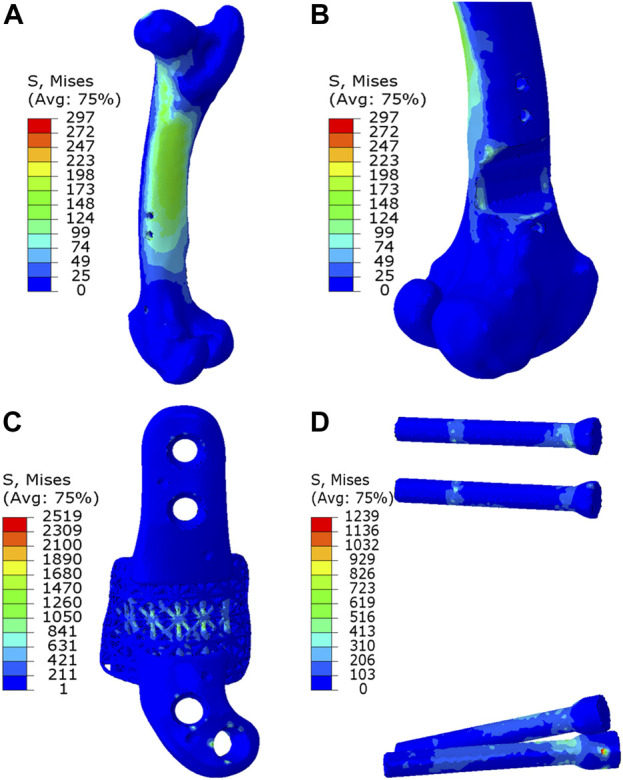
Von Mises stress contour under compression displayed in **(A)** bone, **(B)** bone resection, **(C)** implant and **(D)** screws. The stress unit in the figures is in MPa. In **(B)**, the implant is omitted to enhance visualization of the resected area.

**FIGURE 8:**
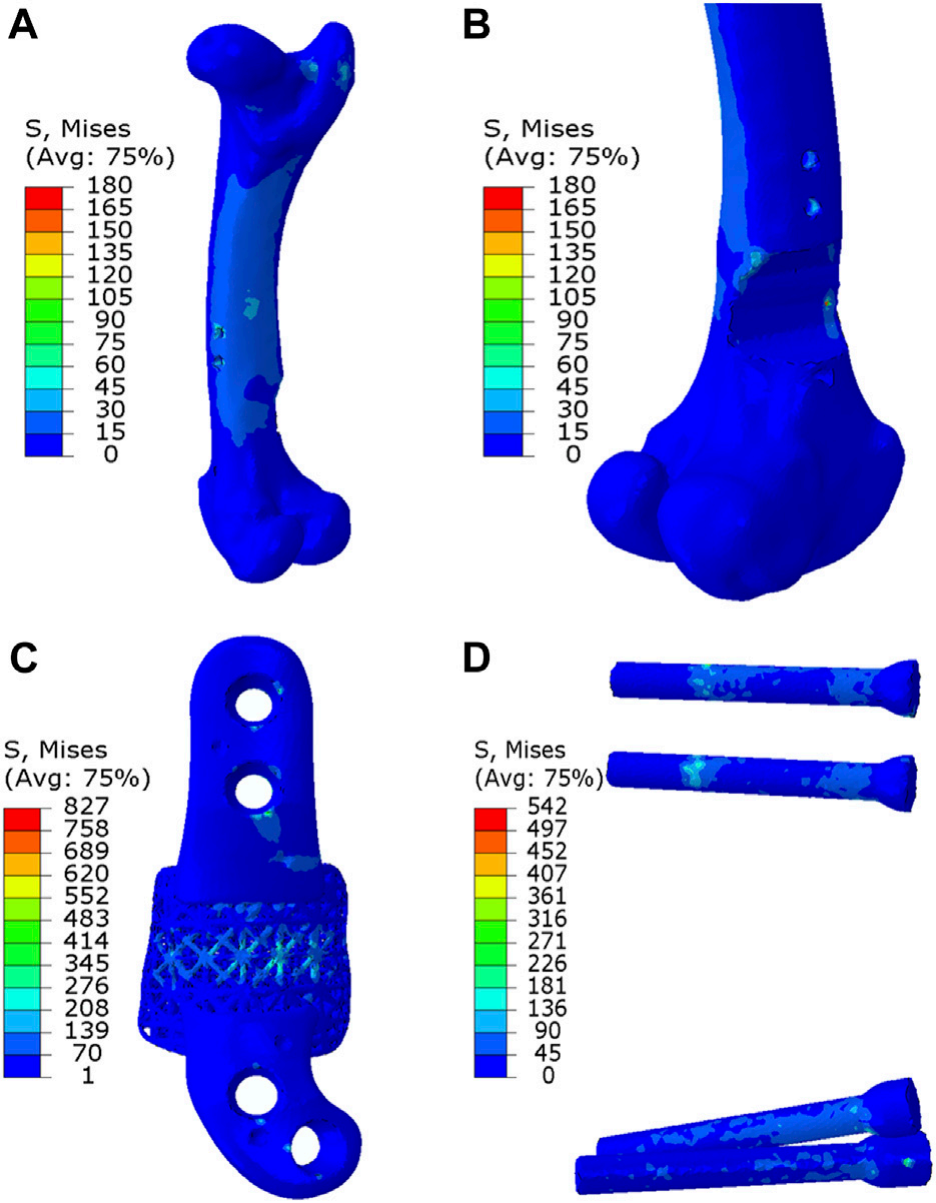
Von Mises stress contour under torsion displayed in **(A)** bone, **(B)** bone resection, **(C)** implant and **(D)** screws. The stress unit in the figures is in MPa. In **(B)**, the implant is omitted to enhance visualization of the resected area.

**FIGURE 9:**
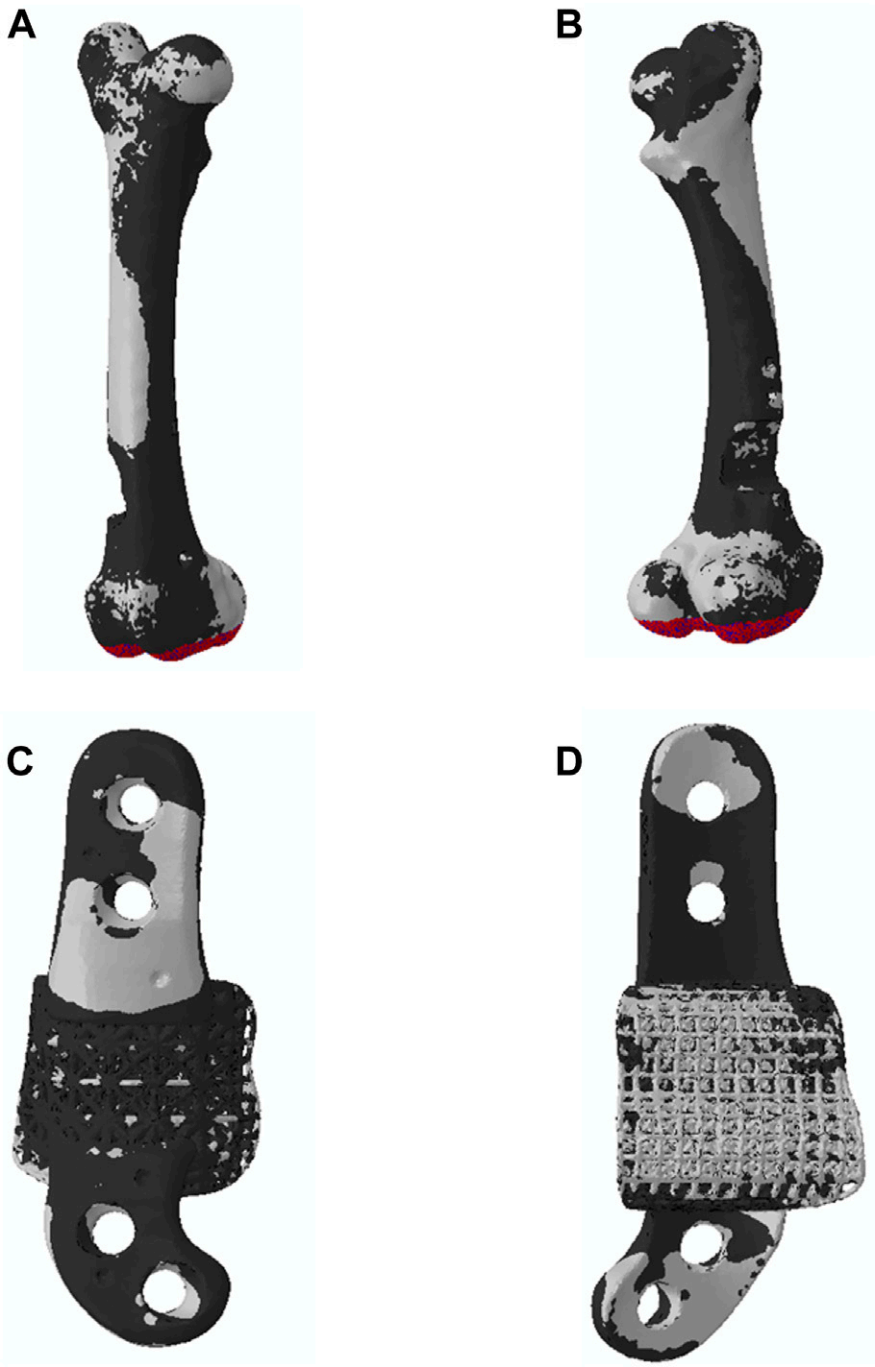
Neutral planes in **(A, B)** bone and **(C, D)** implant in the compression testing, exhibiting the two zones (1) zone under compression in black and (2) zone under tension in white. In the compression testing, the distal extremity was fully fixed, and a downward displacement was applied to the femoral head.

As depicted in Figures 7, 8, the analysis of Von Mises high-stress zones within one of the implanted bone (Sheep #17) indicated that in compression, these areas were predominantly situated in the caudal aspect of the shaft, extending towards the femoral head in a posterior view. Similarly, under torsion loading, these stress zones were predominantly located in the caudal shaft. High-stress concentrations were notably identified in the middle struts of the lattice implant and within the regions where the screws made contact with both the implant and bone. These areas experienced the highest stress levels. In the case of compression testing, it is important to note that due to off-center loading (eccentric compression), the setup experienced not only compression loading but also bending loading, leading to some areas experiencing tension, while most areas were still exposed to compressive stresses. This phenomenon can be explained by the principle of superposition. In light of these complex loading conditions, a thorough analysis was carried out to determine the neutral planes within both the bone and implant. The results of this analysis revealed that different areas within both the bone and implant were exposed to a combination of compression and tension forces. This is illustrated in Figure 9, where neutral planes can be identified.

### 3.3 Sensitivity study

Our proposed FE model was employed to conduct three sensitivity analyses, each of which examined the impact of specific factors on implant performance. The results presented in the following sections are derived from numerical simulations.

#### 3.3.1 Variation in Implant shape and topology

**FIGURE 10:**
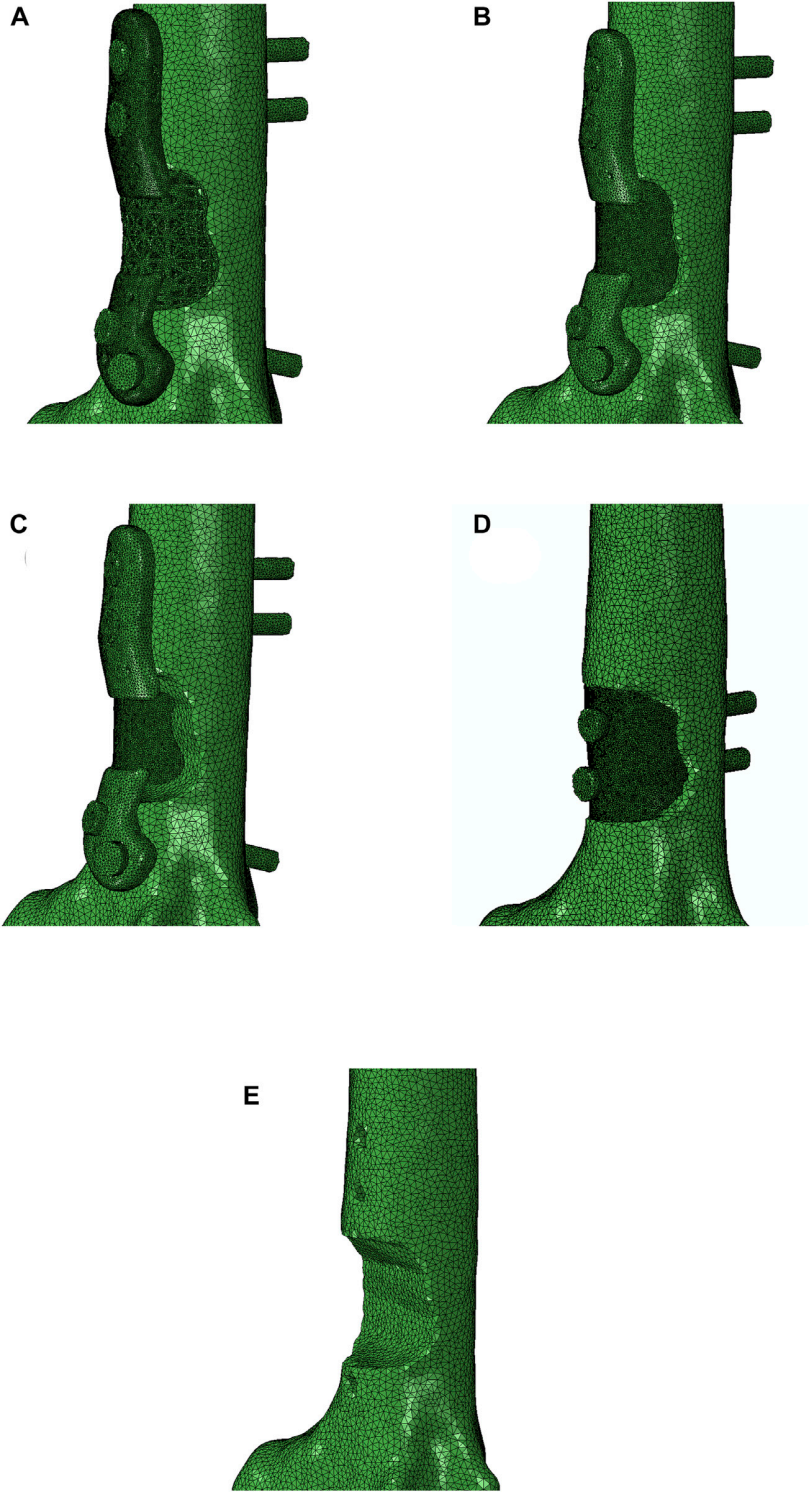
Four different implants were modeled for a comparison: **(A)** lattice implant, **(B)** fully solid implant, **(C)** non-filling implant and **(D)** fully solid implant with no flanges. **(E)** displays the resected bone with no implant for better comparison. The secondary region of the implant is removed in the non-filling implant.

This sensitivity analysis compares the effects of four distinct implant shapes, each represented in Figure 10:

A. Lattice implant: Features lattice structures in both primary and secondary regions, which are attached to solid flanges.
B. Fully solid implant: Exhibits fully solid primary and secondary regions attached to solid flanges.
C. Non-filling implant: Comprises a solid primary region only, attached to solid flanges, with the secondary region removed to serve as a bridging implant.
D. Fully solid implant with no flanges: Consists of fully solid primary and secondary regions without flanges; instead, two screws secure the implant to the bone through both regions.

**FIGURE 11:**
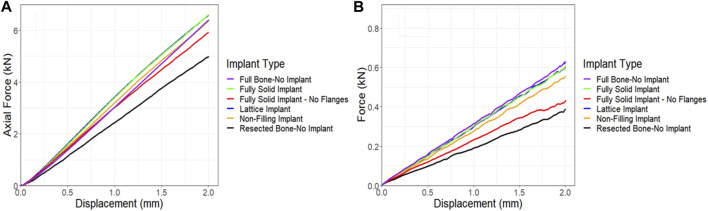
Comparison of force-displacement curves for a sheep femur bone, implanted with four different implants under **(A)** compression and **(B)** torsion. The full bone and the resected bone with no implant states were also included for better comparison.

The compression simulations revealed that the lattice and the fully solid implants produced nearly identical results. The model fitted with a non-filling implant exhibited slightly lower stiffness, approximately 3% less than the ones fitted with the lattice or solid implants. In an overall assessment, all models bearing any of the three implant variations, fully solid, and non-filling, demonstrated stiffness characteristics similar to the control model. Notably, the model fitted with a fully solid implant with no flanges exhibited a lower stiffness, approximately 10% less stiff than the lattice implant bearing model (as depicted in Figure 11A). Similar trends were observed in the torsion simulations, albeit with more pronounced differences. Once again, the lattice and fully solid implant bearing models displayed nearly identical results, closely approximating the stiffness of the control model. In contrast, the model fitted with a non-filling implant was 7.5% less stiff. The most significant variation was observed in the model fitted with a fully solid implant with no flanges, where a 28% lower stiffness was found when compared to the models fitted with the lattice and fully solid implants (Figure 11B).

#### 3.3.2 Friction coefficient sensitivity

**FIGURE 12:**
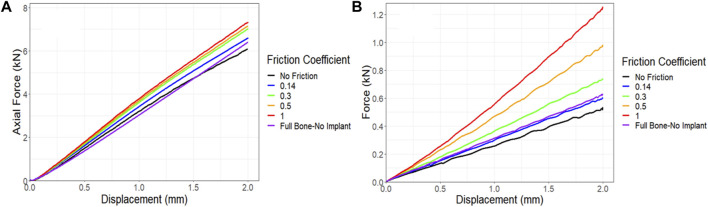
Impact of friction coefficient between bone and the implant and screws on the force-displacement curve for a sheep femur bone, implanted with a lattice implant under **(A)** compression and **(B)** torsion. The full bone state was also included for better comparison.

In all simulations, a friction coefficient of 0.14 was utilized at the contact points. However, due to limitations in measuring the friction between the bone, implant, and screws, an additional sensitivity analysis was conducted to assess the impact of the friction coefficient on force-displacement results and compared these results to those of the control. Figure 12A demonstrates that the friction coefficient of 0.14 produced results that closely matched those of the control. Notably, increasing the friction coefficient from a frictionless state to a no-slip state (from 0 to 1), resulted in a 20% increase in the maximum force in compression simulations. The torsion simulations further supported the conclusion that a friction coefficient of 0.14 represented the optimal choice compared to the control following the 6-month survival period. However, a significant change was observed when the friction coefficient was raised from 0 to 1, resulting in a remarkable 130% increase in the maximum force, as depicted in Figure 12B.

#### 3.3.3 Implant stiffness sensitivity

**FIGURE 13:**
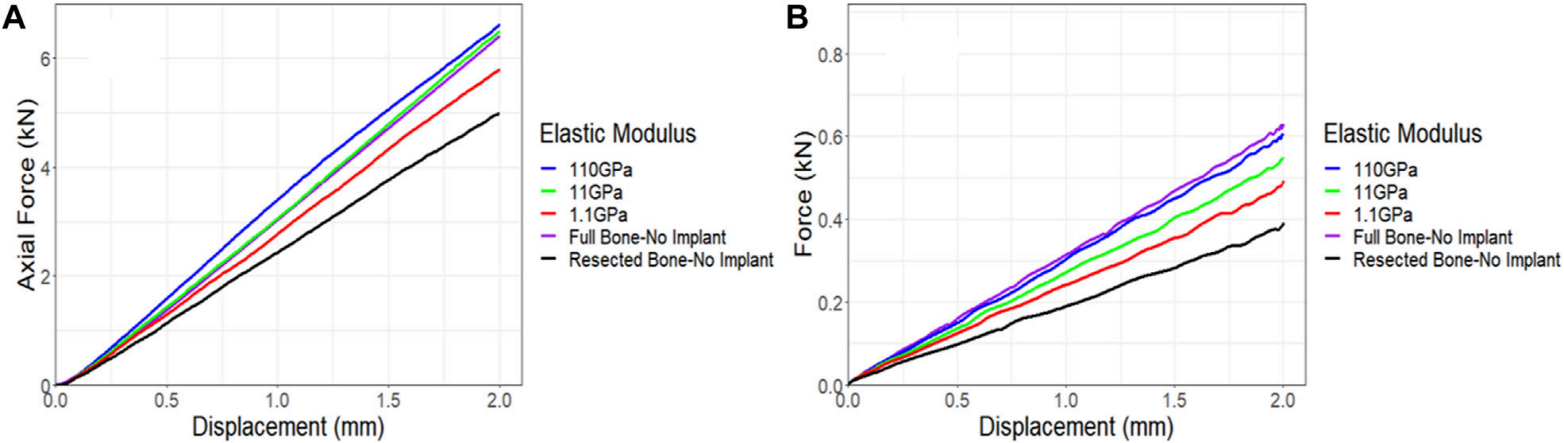
Impact of implant stiffness on the force-displacement curve for a sheep femur bone, implanted with a fully solid implant under **(A)** compression and **(B)** torsion. The full bone and the resected bone with no implant states were also included for better comparison.

Given the absence of a significant difference in stiffness between the models fitted with the lattice and solid implants in both simulations and experiments, a sensitivity analysis was conducted to investigate the impact of implant stiffness on the performance of the overall bone-implant constructs. In all simulations, validated against experimental data, Ti64 was modeled with an elastic modulus of 110 GPa and a Poisson’s ratio of 0.3. In the real world, the elastic modulus of lattice implants can be adjusted by modifying the dimensions and structure of the lattice units or pores (Leary et al., 2018). However, for simplicity in this sensitivity analysis, the elastic modulus of a fully solid implant was systematically reduced, examining the impact of reducing it by 10 and 100 times. The results were then compared to the behavior of the control bone and resected bone without an implant. Figure 13A illustrates that even reducing the elastic modulus by a factor of 10 had a limited effect on the behavior of the implanted bone under compression. However, when the implant had an elastic modulus 100 times lower, it was notably less stiff, exhibiting approximately a 12% reduction in axial stiffness. The torsion simulations showed a similar trend. Decreasing the elastic modulus of the implant by a factor of 10 and 100 resulted in reductions of approximately 9% and 19%, respectively, in the stiffness of the implanted bone (Figure 13B).

## 4 Discussion

In this study, we employed a FE model to predict the stiffness of sheep femora that were reconstructed with endoprostheses and its accuracy when validated against data obtained from quasi-static compression and torsion testing of matched experimental samples harvested 6 months after the implantation procedures in sheep.

Our FE model demonstrated good agreement with experimental results for both the control (non-implanted) and implanted bones. However, we observed some discrepancies in the results for the implanted bones, which can be attributed to a number of factors. One of the primary contributors to these discrepancies was the reliance on preoperative CT scans to extract each bone’s material properties that were used in the construction of the FE models. Thus, our FE simulations did not account for bone ingrowth (osteointegration) and remodeling that occurred within the six-month survival period after which the experimental data were collected.

Based on microcomputed tomography 6 months post-implantation, substantial structural remodeling was observed in and around the implants and particularly beneath the flanges of the solid implants where significant bone resorption activity had taken place. Previous studies have also highlighted the issue of bone resorption beneath solid titanium implants with high Young’s moduli due to stress shielding (Thelen et al., 2004; Black, 2005; Niinomi and Nakai, 2011). In line with these observations, our previous work reporting the microtomographic and histological findings of similarly treated sheep at 3 months, found significant loss of periprosthetic bone integrity in femora that were reconstructed with the solid implants (Sanaei et al., 2023).

Moreover, we observed significant bone ingrowth within the lattice structure of lattice implants, highlighting the advantage of this implant type in facilitating osteointegration. These post-implantation changes, driven by stress shielding and osteointegration, can well explain the discrepancies observed between the simulated and experimental results, particularly in the case of the solid implanted bones.

The FE model provided more realistic results for the bones fitted with the lattice implant compared to those with the solid type suggests minimal bone anatomical and material changes within the 6 months survival period in this group. These findings emphasize the advantages of lattice implants, which not only encourage bone ingrowth but also lead to reduced bone remodeling compared to solid implants, consistent with earlier research (Thelen et al., 2004; Niinomi and Nakai, 2011; Park et al., 2019). Our analysis of correlation generally indicated greater agreement between values predicted by the FE model and those measured experimentally when placed under torsional loading. This finding suggests that the impact of stress shielding, caused by the solid implant in this study, on bone stiffness is more substantial under compression rather than under torsion. This further confirms the previously reported conclusions of our animal study on the effects of prosthesis modulus on bone structure (Sanaei et al., 2023).

The objective of our investigation was to develop a FE model that would enable the design engineer to screen and fine-tune the AM custom endoprostheses throughout the design stage in an effort to prevent the occurrence of acute implant failure following implantation. In this respect, our use of experimental data to validate the model was opportunistic and intended to replace the need for further animal experimentations. Additionally, our model currently does not take into account the effect of fatigue on implant integrity. However, we plan to enhance our FE model by incorporating fatigue analysis, which will be supported by planned fatigue tests in the future.

Given the morphology of the bone and its natural position between the hip and knee joints, the femur typically experiences loading along its mechanical axis rather than its anatomic axis. Our model highlighted the significance of this off-center loading (eccentric compression), demonstrating that it induces both compressive and tensile loadings at different locations in the femur bone. Consequently, the location of the implant plays a critical role in determining the magnitude and direction of stresses within the implant. Therefore, utilizing our FE model can greatly aid in implant design and performance simulation under the corresponding loading conditions.

In this study, we conducted three sensitivity analyses to explore the impacts of (i) implant shape and topology, (ii) friction, and (iii) implant stiffness on the overall construct performance. Our investigations revealed that models fitted with both fully solid and lattice implants exhibited similar axial and torsional stiffnesses. This lack of difference indicates that inclusion of porosity (lattice) into the implant, using design parameters reported here, does not increase the likelihood of acute implant mechanical failure in patients receiving lattice endoprostheses and as such should be clinically utilized more extensively. That said, more research is required to model the effects of metal fatigue and stress shielding in order to achieve greater fidelity in simulations and further insight into this area.

The models fitted with the non-filling implant displayed slightly lower stiffnesses compared to the models fitted with the fully solid and lattice implants. Notably, models bearing the fully solid implant with no flanges exhibited a significantly lower stiffness, particularly under torsion. Likewise, a prior study demonstrated that variations in implant fixation can lead to differing load distributions and stress patterns, subsequently influencing bone ingrowth within the bone (Liu et al., 2022).

The results of the animal study revealed significant bone resorption particularly beneath the solid flanges. These modeling findings suggest that employing a lattice implant with no flanges can reduce implant stiffness greatly, potentially leading to less bone resorption and periprosthetic remodeling. Furthermore, this approach allows for bone regrowth within the implant, reinforcing it over time. While our simulations indicated that removing flanges may decrease torsional stiffness by 28%, our friction sensitivity analysis demonstrated that enhanced integration of the implant into the bone can increase torsional stiffness, aligning with findings from a previous study (Liu et al., 2022). Anticipating stronger bonding between the lattice implant and bone after surgery, due to osteointegration and bone ingrowth, suggests that a lattice implant with no flanges has the potential to achieve substantially higher torsional stiffness over time.

In our sensitivity analysis on implant stiffness, we found that reducing the elastic modulus of a fully solid implant by tenfold did not produce significant changes in implant behavior under compression and torsion, in agreement with a previous study (Yan et al., 2020). However, a remarkable 12% reduction in axial stiffness and a 19% reduction in torsional stiffness were observed when the implant’s elastic modulus was decreased by 100 times. These results imply that employing a fully lattice implant, with lattice flanges featuring larger unit cells and smaller strut diameters, can effectively reduce implant stiffness. This reduction may help minimize bone remodeling and resorption as seen with the solid implants. As discussed, this will have the added benefit of better osteointegration, however, given the effect of lattice design and integrity on osteointegration, this requires further *in vivo* investigation. The lattice pore geometry, in addition to stiffness, significantly influences bone growth (Zhao et al., 2021). Since bone structure is heterogenous, customizing lattice structures based on defect location can optimize bone ingrowth (Metz et al., 2020). Integrating mechanobiological optimization algorithms (Rodríguez-Montaño et al., 2019; 2020; Wu et al., 2021; Perier-Metz et al., 2022) with our FE model provides a comprehensive toolkit for designing structurally sound lattice implants. This approach minimizes stress shielding and maximizes bone ingrowth in alignment with natural bone structure.

## 5 Conclusion

In this study, we conducted a comprehensive finite element analysis of patient-specific AM implants used in the endoprosthetic reconstruction of ovine femur. The research aimed to assess the performance of two types of solid and lattice implants after phantom bone tumor resection and their impact on the overall stiffness of bone-implant construct.

Our findings highlighted the efficacy of the proposed FE model in accurately predicting the mechanical responses of implanted bones. Validated against experimental data from an animal study involving nine sheep, the model demonstrated its capability to simulate and assess various implant designs, offering an effective tool for the purpose of optimizing implants tailored to individual patient needs.

Notably, our investigations indicated that reducing the elastic modulus of solid implants by tenfold had minimal effects on bone behavior under compression and torsion loadings. This highlights the remarkably high stiffness of solid titanium implants. Consistently, employing fully lattice implants demonstrated similar advantages with the added benefit of promoting bone ingrowth and minimizing stress shielding. Our findings indicate that increased friction between the implant and the bone, facilitated by stronger osteointegration, greatly enhances the overall stiffness of the implanted bone. Consequently, the utilization of a lattice implant with lower stiffness, in comparison to a solid implant, can potentially confer adequate strength to the bone once osteointegration is underway.

Overall, our research underscores the potential of additive-manufactured implants, especially lattice structures, in facilitating natural bone healing and minimizing adverse effects on surrounding bone tissues. These insights help pave the way for the future advancement of patient-specific implant design, emphasizing the importance of optimizing implant properties for improved patient outcomes.

Further studies involving larger sample sizes and longer-term *in vivo* as well as *ex vivo* assessments are warranted to validate these findings and facilitate the development of more effective implant solutions for bone tumor resection and better bone ingrowth.

## Data Availability

The raw data supporting the conclusion of this article will be made available by the authors, without undue reservation.
